# Electrochemical Instrumentation of an Embedded Potentiostat System (EPS) for a Programmable-System-On-a-Chip

**DOI:** 10.3390/s18124490

**Published:** 2018-12-18

**Authors:** Adrián Iván Muñoz-Martínez, Omar Israel González Peña, Jordi Colomer-Farrarons, José Manuel Rodríguez-Delgado, Alfonso Ávila-Ortega, Graciano Dieck-Assad

**Affiliations:** 1 Tecnologico de Monterrey, School of Engineering and Sciences, Av. Eugenio Garza Sada Sur No. 2501, Col. Tecnologico, Monterrey 64849, Mexico; adrian.ivan.munoz@gmail.com (A.I.M.-M.); jmrd@itesm.mx (J.M.R.-D.); aavila@itesm.mx (A.Á.-O.); graciano.dieck.assad@itesm.mx (G.D.-A.); 2 Department of Electronics and Biomedical Engineering, Bioelectronics and Nanobioengineering Research Group (SIC-BIO), University of Barcelona, Martí i Franquès, 08028 Barcelona, Spain; jcolomerf@ub.edu

**Keywords:** chronoamperometry, potential sweep methods, reconfigurable embedded potentiostat, portable potentiostat, programmable-system-on-a-chip, wireless electronic

## Abstract

Under the main features required on portable devices in electrochemical instrumentation is to have a small size, low power consumption, economically affordable and precision in the measurements. This paper describes the development of a programmable Embedded Potentiostat System (EPS) capable of performing electrochemical sensing over system-on-a-chip platforms. Furthermore, the study explains a circuit design and develops some validation of the entire system. The hardware validation is performed by electrochemical experiments such as Double Step Chronoamperometry (DSC), Linear Sweep Voltammetry (LSV) and Cyclic Voltammetry (CV); moreover, a comparison of the experimental signals between a commercial potentiostat and the EPS was done by analysis of errors on the response signal. Results illustrate that the EPS is capable of handling currents in the range of absolute values of 86.44 to 3000 nA and having control voltages in the range of ±2 V. The device can support from 50 to 2000 samples per second. The EPS capabilities were compared with other compact potentiostats. The programmable EPS is an original approach which hugely reduces the hardware complexity and leads the way to create new applications for Point-of-Care or industrial developments with a reusable full electronics module.

## 1. Introduction

A potentiostat is a device which can input predetermined voltage/current signals that generate outputs with an electron-related behavior needed to study redox reactions [1]. The potentiostat also relies on a feedback loop usually implemented with advanced electronic components to accurately control and condition electrical potential differences obtained from sensors to ensure reliable information at the output.

Before the invention of computers to control voltage and/or current, it was challenging to obtain signal processing in electrochemical instrumentation. Indeed, modern electroanalytical chemistry started with the invention of polarography in the 1920s [2]; since then, the electrochemical instrumentation has been pushing forward according to progress achieved in electronics (Moore’s law) and information technology (Internet of Things). Incorporation of electrochemical sensors continues to gain a presence in research efforts to develop technology in different application fields such as Lab-on-a-chip devices or centrifugal microfluidic platforms [3]; indeed, these microfluidic platforms have proved to be very convenient for clinical diagnosis of glucose and cancer cell detection issues [4,5,6].

Point of Care Technology (POCT) devices make possible to obtain sample measurements of patients by using wireless communication and under a large distance between patients and professionals of health by using the internet of things technology. This technology offers features such as shortening the sample analysis periods, reducing the size of the final device reaching portability. Thus, it is possible to implant POCT devices in humans for continuous monitoring purposes [7,8].

Trends in microelectrode fabrication, microfluidics and microelectronic systems have resulted in both challenges: in the design/development of potentiostats and significant advances in the capabilities of the potentiostats to collect data at the transient that take place at different time constants associated to a different phenomenon; for instance, in the order of ~(10^−15^ to 10^−11^) s, it is possible to observe the ohmic drop of the system, or if a experimentalist will like to observe the time constant associated with molecular diffusion it is required to record the electrochemical signal of the experiment in the order to ~(10^−3^ to 50) s [9]. Consequently, in the middle on the mass transfer process and the ohmic drop of the system occurs the time constant associated with electrochemical reactions [10]. As a result, the potentiostats have become very sophisticated systems to make possible applications such as DNA identification, protein classification, neural recording, glucose-level determination, PH detection, drug-concentration quantification, among others.

The lack of the information about the circuitry is one of the most important disadvantages present in commercially supplied potentiostats [8,10,11,12,13]. This information related to electrochemical detection is necessary to manipulate certain variables like the voltage waveforms. Thus, experimentalists have to adapt the methodologies under development to the available potentiostats in the market; likewise, the lack of this information results in different kinds of problems to develop new measurement approaches for the need for highly customized and flexible electrochemical instruments for hardware and software.

**Figure 1:**
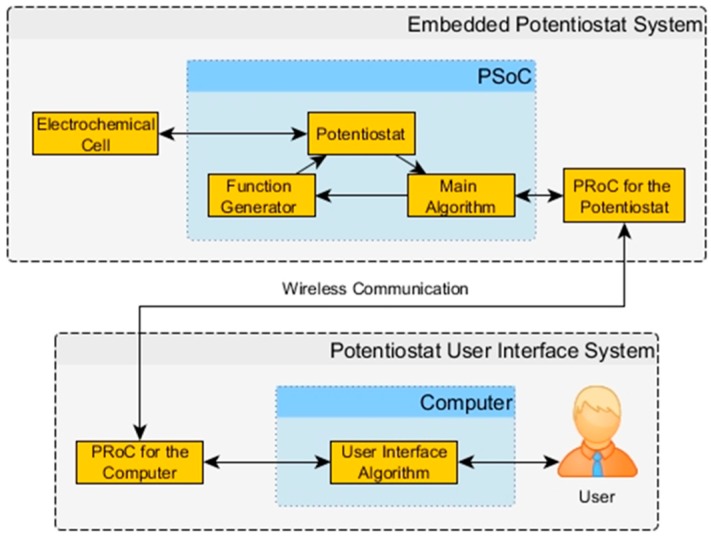
Block Diagram of the Embedded Potentiostat System (EPS) with its User Interface.

This study had proposed a highly customizable and flexible platform consisting of the electronic circuits and the software to drive redox reactions. In addition, it is presented in the study a well-description and characterization on the potentiostat system, which it is necessary for making possible the availability of technological devices [8,10,11,12,13,14]. Figure 1 shows a possible solution to have a highly customized potentiostat system for applications like Lab-on-a-System, wearable monitoring systems and POCT. The idea is to have an embedded system small enough to meet the application requirements. In this case, the Programmable System on a Chip (PSoC) and the Programmable Radio on a Chip (PRoC) have shown their worth [14,15,16,17]. The interface system uses LabVIEW in a computer to deal with the user with a versatile graphical environment [18,19,20,21,22]. The communications between computer and PSoC is wireless. The scalability of the system takes place using pattern designs at the software level.

Furthermore, it is presented in this work a flexible and integral methodology that includes the characterization and calibration of the potentiostat, thus the electronics of the device were tested by performing three electrochemical techniques and its analysis of errors. This methodology allows for the reconfiguration of the device to execute different electrochemical techniques allowing a correct functioning of the equipment.

### 1.1. Background

A potentiostat by itself just controls the potential in an electrochemical cell and more electronic components are necessary to get more information about the electrochemical phenomenon [23,24]. The Digital to Analog Converter (DAC) provides the control signal for the potentiostat. The current measurement circuit reads the electrons flow of the reactions. The Analog to Digital Converter (ADC) turns the analog current values in digital. Thus, the basic potentiostat system defines the performance of the entire instrument.

The function generator can give the waveform values in an analog or digital way. However, a computer generates the digital signals most of the time. Also, the recorder system has to handle digital values because it is the easiest way to save data. The display system can be any device capable of showing information. Though, one of the fastest is a screen. Hence, all these components and the basic potentiostat system let us have a complete device to perform electrochemical experiments.

#### 1.1.1. Embedded Potentiostat System (EPS)

The EPS prototype includes some modifications to incorporate additional electrochemical techniques. The main aspects are the description of the embedded systems and design patterns for programming. An embedded mixed-signal architecture deals with applications where the acquisition, processing and manipulation of the variables are necessary [25]. In a potentiostat system, the electrochemical reaction current is the variable to sense, the voltage at the electrodes is the variable to control and the microcontroller algorithm generates the appropriate waveforms for an electrochemical trial. Thus, a potentiostat application matches with an architecture like this. The main functions to perform by this kind of embedded system are [26]:

- Sensing the analog signals.
- Transmission and reception of data inside and outside of the embedded system.
- Firmware execution.
- Actuation signals generation.

The PSoC from Cypress Semiconductor is one of the icon devices in an embedded mixed-signal architecture [27]. The selection of this device relies on the incorporation of several features in a single chip. Hence, analog, digital and processing systems are inside of a PSoC with the capacity to address several applications. The main feature of the PSoC is its configurability. That allows to an experimentalist to have new solutions to the most challenging problems [15,16,17,18].

A Full potentiostat system, requires the management of analog and digital signals. A PSoC provides an architecture for the treatment of mixed-signals in one chip [17]. These features bring advantages as fast development times, space reduction and simplification of the application.

#### 1.1.2. Prototype Implementation and Architecture

**Figure 2:**
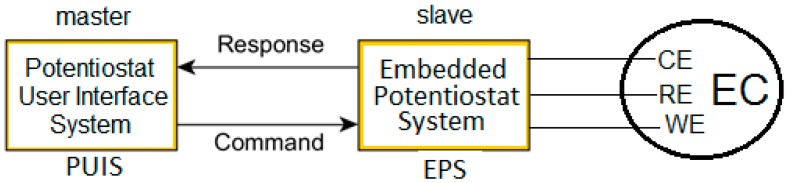
Slave-master scheme: Embedded and User Interface systems (EPS and PUIS) driving the electrochemical cell.

The potentiostat instrument prototype has two main systems: the embedded and the interface system as shown in Figure 2. The EPS is responsible for the manipulation of the electrochemical cell sending and receiving data wirelessly. The Potentiostat User Interface System (PUIS) deals with the user and controls the EPS behavior. Both systems constitute a Master/Slave design where the EPS is the slave and the PUIS is the master.

The modular design allows the completion of the research objectives by using a small embedded system as a slave [27]. Also, the modular design is an excellent feature to customize the system. To implement the potentiostat instrument prototype, the master sends commands to the slave and the slave returns the task response. In this prototype, there is a command for each electrochemical method and the response is a lot of digital values from the trial. The PUIS focuses on managing the recording and display system, on generating the appropriate waveforms and on sending Redox current/voltage values (according to the test) to the PUIS. This multiprocessing capability allows achieving a full potentiostat system. 

**Figure 3:**
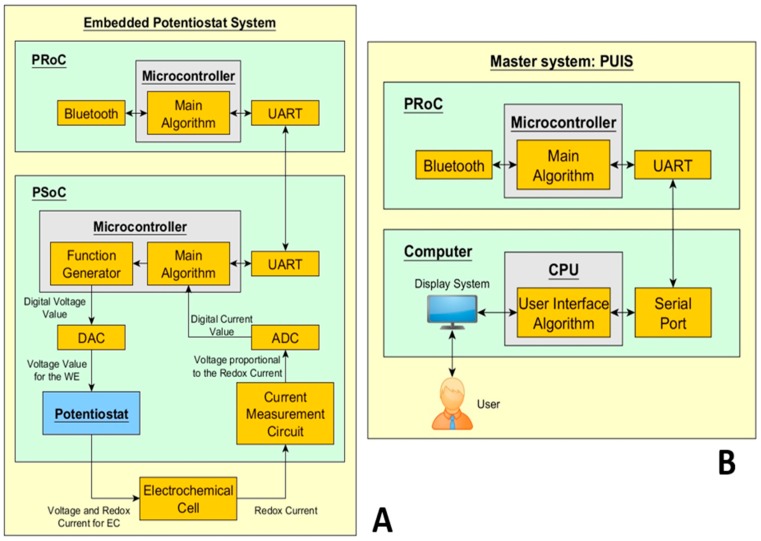
Modular Potentiostat: (**A**) Slave system of the EPS. (**B**) Master system with the structure PUIS.

The EPS is a set of electronic components small enough to be embedded in a potentiostat system application. This system has three main aspects: Bluetooth, PSoC and the electrochemical cell as shown in Figure 3A. The PSoC has analog and digital modules to implement as a potentiostat, a microcontroller and it executes the firmware. The PSoC analog hardware allows to the designer the development of a basic potentiostat system with all the electronic components needed by the typical potentiostat system. Moreover, the microcontroller has the code for the generation of the waveforms according to the electrochemical technique selected and the parameters to stop a running experiment. The main algorithm of the PSoC has a State Machine design pattern programmed in C language. This design pattern is highly acceptable by programmers because its implementation is very flexible and easy to follow [28]. Also, the modularity of the pattern makes feasible the additions of states to implement more electrochemical methods in the same PSoC. Thus, the state machine is an excellent choice to have a friendly firmware because it is very explicit.

Besides the PSoC, Figure 3A shows another chip named PRoC that focuses on Bluetooth communication instead of hardware modules. The addition of this device increases the prototype size. However, this element is extremely important for a successful EPS functionality. Also, the PRoC is one of the best options considering that it comes from the same manufacturer of the PSoC.

The PUIS schematic of Figure 3B uses Bluetooth communication to send commands to the slave. The commands come from a computer with a program and the PUIS is always waiting for any events at the interface to start the electrochemical experiment. Also, it has a recording system to save the data to a computer. Figure 3B shows that the Bluetooth device is out of the computer and it controls all the communication of the master. Moreover, the Bluetooth version is the BLE 4.2 with a data rate up to 25 Mbps for this prototype [29]. The Bluetooth communication works basically as a bridge allowing the design of a simple communication protocol for sending commands. Thus, it is easy the manipulation of bytes to send tasks to the slave and receive the voltage and the current values. 

Recording on a display system is a challenge for any designer. However, the use of advanced design patterns is very helpful. Thus, LabVIEW allows the creation of user interface systems with advanced programming techniques. The algorithm performed by the computer uses a Producer/Consumer and a State Machine design pattern. A later subsection provides more information about these techniques. 

#### 1.1.3. Analog and Digital Circuits in the PSoC

**Figure 4:**
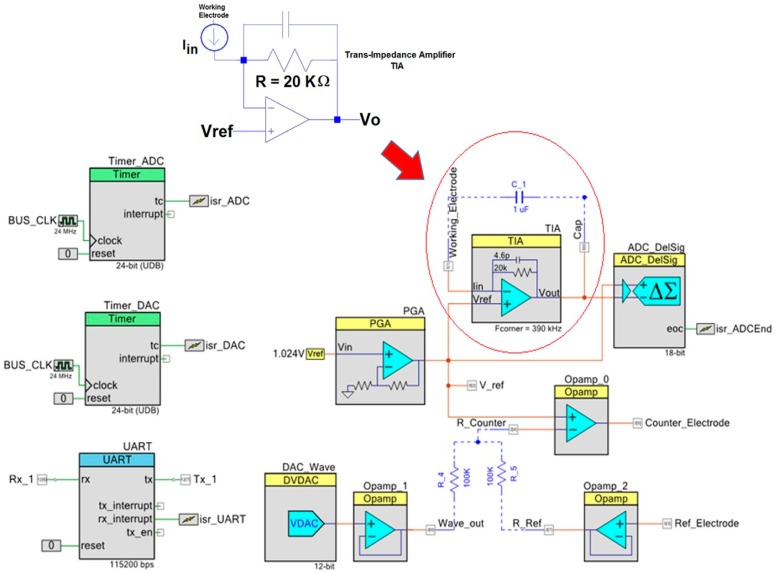
Digital peripherals and analog front end devices of the PSoC. Digital to Analog Converter (DAC), Analog to Digital converter (ADC), Input-Output Port (UART), Trans-Impedance Amplifier (TIA).

The Figure 4 shows the digital schematic circuit of the PSoC, which provides a firmware execution in real-time and the communication to the PRoC. The advanced potentiostat circuit with a Trans-Impedance Amplifier (TIA) from Figure 4 has features used to make the electrochemical prototype tests [1,10]. Moreover, the design of the analog circuitry is very important to provide a good performance in the prototype system. The analog hardware from Figure 4 relies on the advanced potentiostat circuit with the TIA and it has some extra features. The Operational Amplifiers marked as Opamp_0 and Opamp_2 control the potential at the WE through the RE, Opamp_1 supplies the energy for the waveform while the DAC throws the waveform values at the proper rate. The Universal Asynchronous Receiver-Transmitter (UART) module communicates with the Bluetooth module to send data and receive commands wirelessly. The Programable Gate Array (PGA) provides a reference voltage of 2.048 V because the RE can be just manipulated in a range of 0 to 4.08 V. Hence, this floating potential gives a chance to work with ±2 V approximately in the electrochemical cell. The TIA and the ADC transform the current into digital values. The DAC brings some restriction to the embedded application. The maximum quantization error is 0.5 mV because every step is of 1 mV. The minimum time for the DAC to change a value at its output is of 4 µs. Hence, the maximum scan rate for the prototype is 250 V/s in a range of 0 to 4.08 V. The DAC needs the digital waveform value in 12 bits to make the conversion. Also, the DAC requires a buffer at the output to keep the right potential and supply the energy to the potentiostat control signal. 

The TIA module, which it is shown at the top of Figure 4 and the Delta-Sigma Analog to Digital Converter (∆∑ ADC) define the sensitivity of the current measurement. The TIA has eight resistors to have eight different quantization levels. The maximum current value is obtained by using the values for the operation, thus it comes from the minimum resistor of the TIA module (20 kΩ) and the ADC voltage range at its input (±1.024 V) as the equation one describes. However, the missing data needs to be calculated through a characterization.

(1) $$I=\frac{V_{\mathrm{ref}}}{R}=\frac{\pm 1.024\;V}{20000\;\Omega }=\pm 51.2\;\mu A$$

The ∆∑ ADC has several features that define the behavior of this module in the prototype. The conversion mode is a single sample. The ADC has 18 bits and it takes 414 µS approximately to perform one conversion. The clock frequency is around 3071 kHz but the output rate is slower because it uses oversampling to get a better signal quality. The input range is ±1.024 V and the ADC has a buffer to avoid any measurement error by impedance mismatching.

## 2. Materials and Methods

### 2.1. Electrochemical Equipment

**Figure 5:**
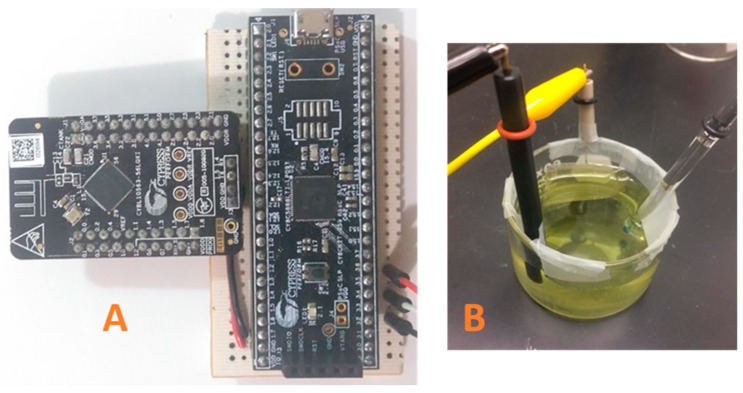
(**A**) Prototype of the EPS. (**B**) Electrochemical cell (height = 35 mm, diameter = 60 mm), solution volume of 50 mL of K_3_[Fe(CN)_6_] and KCl.

The EPS uses two kits as it is shown on Figure 5A from Cypress Semiconductors: CY8CKIT-059 and CY8CKIT-042-BLE, the CY8CKIT-059 kit has the chip CY8C5888LTI-LP097; the CY8CKIT-042-BLE kit has four devices but the prototype just needs the PRoC and the USB dongle. The measurements of the EPS are compared to a commercial potentiostat system (CH Instruments, model 700E). The EPS was operated to recording 2000 data per second which is the maximum samples that the equipment can measure. The three cables on Figure 5A at the bottom right part of the protoboard were connected to the three electrodes of the electrochemical cell on Figure 5B.

### 2.2. Analyte, Electrolyte and Electrodes

All experiments were performed on a volume of 50 mL on a electrochemical cell (height = 35 mm, diameter = 60 mm) of potassium ferricyanide $K_{3}{\left[\mathrm{Fe}(\mathrm{CN}\right)}_{6}]$; this analyte is common to use to test potentiostats [12,30,31,32], since its kinetics is well known and it describes an electrochemical reversible behavior [23,33,34]. Ferricyanide can be reduced to ferrocyanide as Equation (2) shows; the backward direction of the reaction corresponds to the ferrocyanide oxidation to ferricyanide as Equation (3) describes.

(2) $$\mathrm{Fe}{\left(\mathrm{CN}\right)}_{6}^{3-}{+e}^{-}\to \mathrm{Fe}{\left(\mathrm{CN}\right)}_{6}^{4-}$$

(3) $$\mathrm{Fe}{\left(\mathrm{CN}\right)}_{6}^{4-}\to \mathrm{Fe}{\left(\mathrm{CN}\right)}_{6}^{3-}{+e}^{-}$$

In the experiments two analyte concentrations of 1 mM and 10 mM of K_3_[Fe(CN)_6_] (Sigma-Aldrich, Saint Louis, MO, USA, CAS: 13746-66-2) were used to evaluate the EPS. The electrolyte support used was 0.5 M KCl (Fermont, presentation no. 24842). The reference electrode (RE) used is Ag/AgCl (BASi model MF-2052). A platinum wire (BASi model MW-4130) and a disk glassy carbon electrode (BASi model MF-2012, diameter ϕ = 3 mm) was used as the Counter Electrode (CE) and the Working Electrode (WE), respectively, as it is shown in Figure 5B.

### 2.3. Experimental Design

The electrochemical techniques used in the EPS are LSV, CV and DSC. Before comparing the commercial potentiostat with the EPS, the WE were cleaned by immerse it in 0.1 M of HNO_3_ (Sigma Aldrich) for approximately 10 min, later the WE was rinsing with distilled water; after that, the WE received an electrochemical pretreatment to activate its surface by running a sequence of different scan rates of CV and by using 0.1 M of HCl (Sigma Aldrich); The CV sequences of the activation surface is shown on Table 1.

The process of surface activation is initiated with a high scan rate (500 mV/s) and through lower scan rates until a scan rate of (50 mV/s) is reached. In the process of surface activation, all CVs were done on the windows of scan potentials of (−0.15 to 0.65) V versus Ag/AgCl where the initial voltage was set at 0.25 V versus Ag/AgCl. Likewise, the number per cycles of each sequence of CV is higher (50 cycles) at the highest scan rate and it decreases at lower scan rates until reaching (5 cycles). All experiments were carried out at room temperature ~25 °C and the potential recorded was against the Ag/AgCl saturated.

The Randles-Sevcik equation presented below relates to the scan rate, the molecular diffusion and the bulk concentration of the analyte with the current peak from a CV or LSV experiments [34,35].

(4) $$I_{p}=\left(2.69\times 10^{5}\right)n^{3/2}{\left(\mathrm{Dv}\right)}^{1/2}{\mathrm{AC}}^{\mathrm{bulk}}$$

Here, $I_{p}$ is the maximum current (A), n is the number of electrons per mole oxidized or reduced, D is de diffusion coefficient (cm^2^/s), v is the scan rate of the CV or LSV (V/s), A is the working electrode area (cm^2^) and $C^{\mathrm{bulk}}$ is the bulk concentration of the oxidized or reduced specie (mol cm^−3^). Table 2 shows the parameters of the Randles-Sevcik equation and the current peaks of the two concentrations tested in an ideal Nernstian reversible system and under the assumption of semi-infinite linear diffusion. 

The setup condition for each experiment was related to the number of experimental conditions. The conditions rely on the previous investigation where similar values were used [10,14]. The only changes between conditions were the scan rate value. Hence, conditions allow us to evaluate the EPS at different currents magnitudes and scan rates. In addition, a comparison was done of the EPS signal with a commercial potentiostat. Table 3 describes the setup parameters for the conditions tested on CVs. 

Table 4 describes the conditions for the DSC experiments. The small changes between the first and the last step allow us to explore the changes in the current measurements on the prototype and it can be related to the lowest limit of detection on the device. The pulse width was set to 62 s since at that time the current measurement reaches the steady-state response. The last step is practically the open circuit potential of 0.308 V for 1 mM K_3_[Fe(CN)_6_] in Table 4. 

Table 5 describes the conditions for the LSV experiments. The conditions rely on the previous investigation where similar values were used [10,14], where the only changes were the scan rates. 

## 3. Results and Discussion

**Figure 6:**
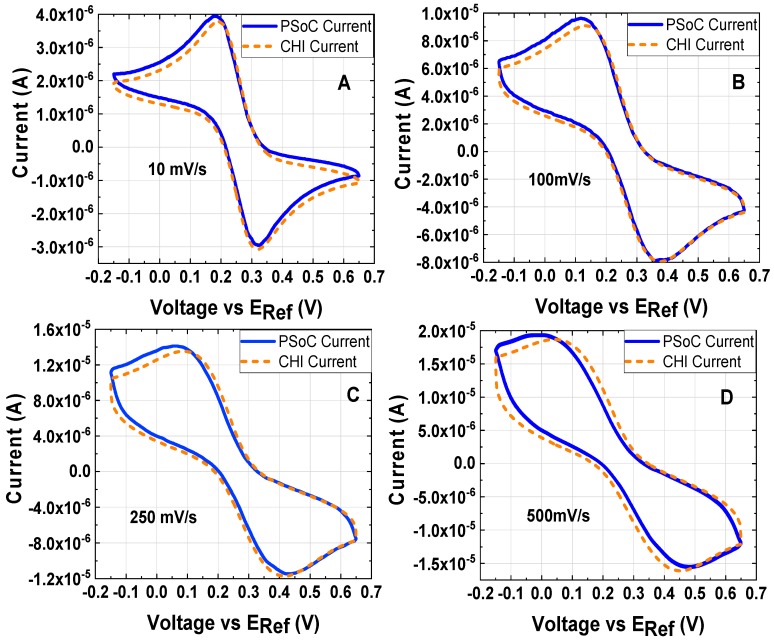
Cyclic voltammetry at different scan rates of 1 mM K_3_[Fe(CN)_6_] and 0.5 M KCl as the electrolyte support; the working electrode, the counter electrode and the reference electrode were a disk glassy carbon electrode (diameter ϕ = 3 mm), a platinum wire and Ag/AgCl electrode, respectively: (**A**) 10 mV/s (experiment under the conditions 1). (**B**) 100 mV/s (experiment under the conditions 2). (**C**) 250 mV/s (experiment under the conditions 3). (**D**) 500 mV/s (experiment under the conditions 4).

**Figure 7:**
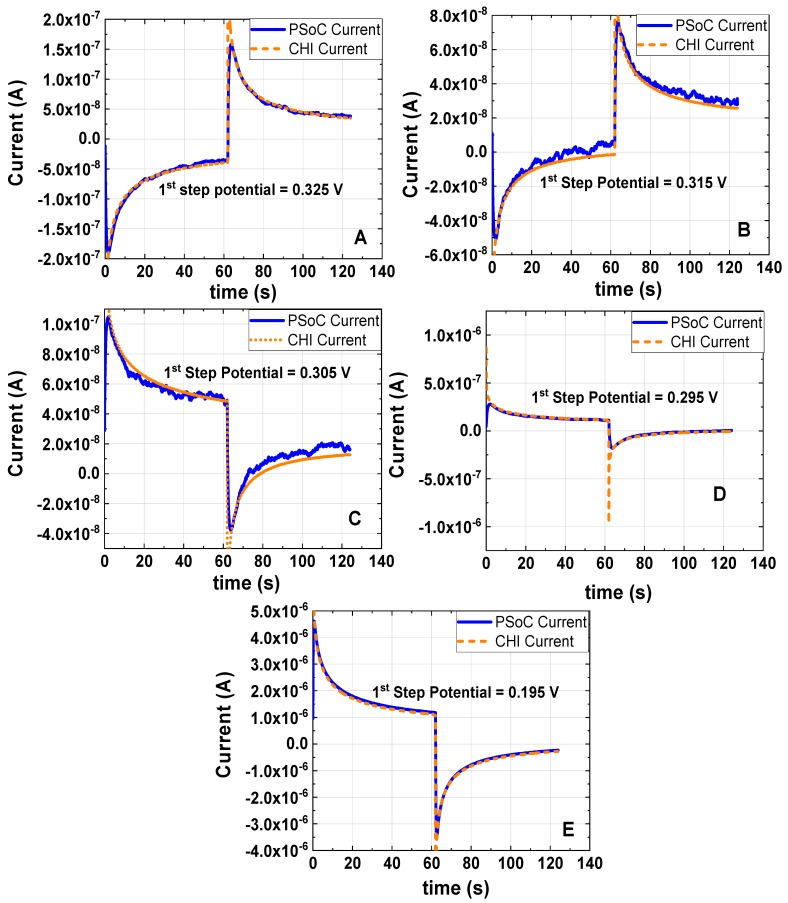
Double Step Chronoamperometry at different first step potentials of 1 mM K_3_[Fe(CN)_6_] and 0.5 M KCl as the electrolyte support; the working electrode, the counter electrode and the reference electrode were a disk glassy carbon electrode (diameter ϕ = 3 mm), a platinum wire and Ag/AgCl electrode, respectively. All DSC were done with a pulse width of 62 s and the last step was setup at 0.310 V vs. Ag/AgCl: (**A**) first step potential 0.325 V vs. Ag/AgCl (experiment under the conditions 5). (**B**) first step potential 0.315 V vs. Ag/AgCl (experiment under the conditions 6). (**C**) first step potential 0.305 V vs. Ag/AgCl (experiment under the conditions 7). (**D**) first step potential 0.295 V vs. Ag/AgCl (experiment under the conditions 8). (**E**) first step potential 0.195 V vs. Ag/AgCl (experiment under the conditions 9).

**Figure 8:**
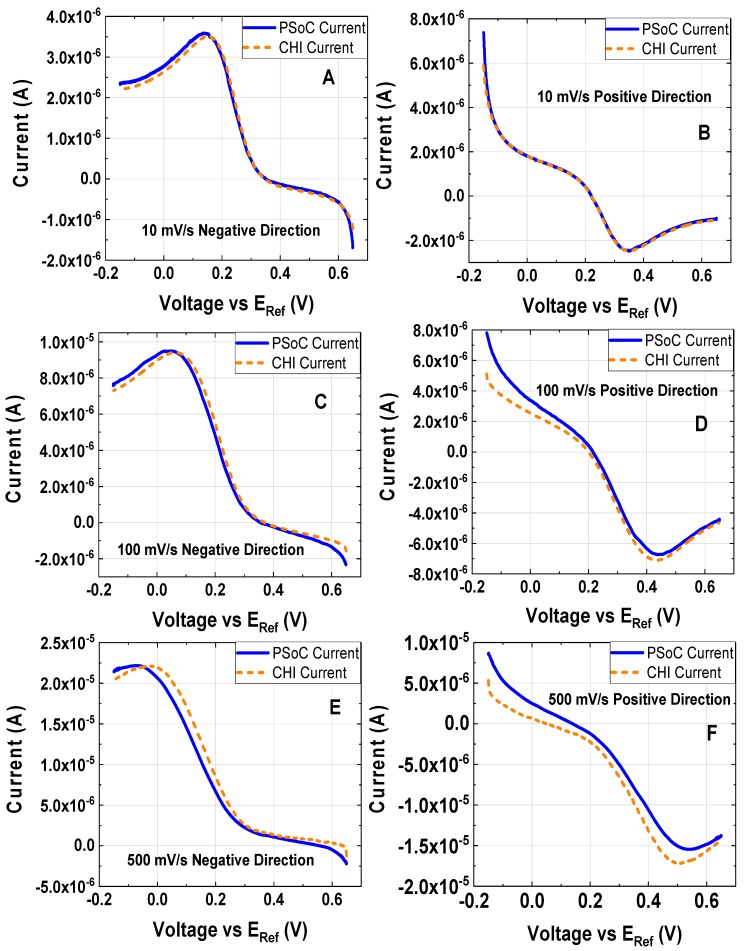
Linear sweep voltammetry at different scan rates at different first step potentials of 1 mM K_3_[Fe(CN)_6_] and 0.5 M KCl as the electrolyte support; the working electrode, the counter electrode and the reference electrode were a disk glassy carbon electrode (diameter ϕ = 3 mm), a platinum wire and Ag/AgCl electrode, respectively. All voltages are reported vs. Ag/AgCl. (**A**) Scan rate 10 mV/s, initial and final voltage are 0.65 V and −0.15 V, respectively (experiment under the conditions 10). (**B**) Scan rate 10 mV/s, initial and final voltage are −0.15 V and 0.65 V, respectively (experiment under the conditions 11). (**C**) Scan rate 100 mV/s, initial and final voltage are 0.65 V and −0.15 V, respectively (experiment under the condition 12). (**D**) Scan rate 100 mV/s, initial and final voltage are −0.15 V and 0.65 V, respectively (experiment under the conditions 13). (**E**) Scan rate 500 mV/s, initial and final voltage are 0.65 V and −0.15 V, respectively (experiment under the conditions 14). (F) Scan rate 500 mV/s, initial and final voltage are −0.15 V and 0.65 V, respectively (experiment under the conditions 15).

Results of the experimental conditions in Table 3, Table 4 and Table 5 are shown in the graphs from Figure 6, Figure 7 and Figure 8. In the LSV and CV, the voltage values are versus the RE and it is indicated as EREF on the abscissa axis. All electrochemical experiments follow the sign convention used on the commercial potentiostat (chemistry convention); therefore, the peak currents observed on CVs in Figure 5 with negative magnitude, correspond to the oxidation in the Equation (3); contrary, the positive magnitude of the current corresponds to a reduction in the Equation (2). A minor discrepancy on the signal of the prototype occurred at high currents; however, the results from the prototype are close to the commercial potentiostat in most of the graphs when it is considered a proper range to work for the prototype. A minor drawback of the potentiostat prototype is the filter; this capacitor introduces a shift phase and it is possible to been observed when the scan rate is fast as Figure 6D shows. This filter is necessary because noise appears in the measurements specially when very low currents are monitored. Thus, this component allows us to reduce the detection limits sacrificing a little of the potentiostat prototype bandwidth.

In Figure 7, it is shown different DSCs at different first step potentials described in Table 4. In Figure 7A,B, the initial step corresponds to the oxidation and on the second step it is shown a reduction; contrary, in Figure 7C–E) the process has been inverted. 

In Figure 7B,C, the current recorded at longer times describes more evidently an oscillation when the system is close to reach a relaxed response; This oscillation can be related to the perturbation step signal, which was very close to the open circuit potential; as a result, in Figure 7B,C, the ratio of the peak currents divided by the current measured at the steady-state provides a less abrupt ratio compared to when the system is under a large perturbation signal of a given step of potential. 

Figure 8 shows the LSV experiments described in Table 5. In Figure 8A,C,E, it is shown that LSV under a cathodic scan corresponds to a reduction; on the other hand, Figure 8B,D,F shows the anodic direction on the LSV associated with an oxidation. A little discrepancy of the phase response was observed at the maximum scan rate of 500 mV/s for the cathodic and anodic directions with respect to the commercial potentiostat response; the small difference can be associated with the same signal observed at the highest scan rate of the CV experiment. 

### Analysis of Results

An error analysis will show the differences between both devices for each experiment quantitatively. The absolute error express how far is the measured value of the real as the Equation (5) describes. In these experiments, the real values are from the commercial potentiostat while the measured values are from the prototype. The mean error refers to the average of the absolute errors in an experiment as Equation (6) agrees. The highest error is a value very close to the maximum error because it comes from the standard deviation (σ) of the absolute errors as Equation (7) shows.

(5) $$\mathrm{Absolute}\;\mathrm{Error}\;=\;\left|\left|{\mathrm{Value}}_{\mathrm{Measured}}\right|-\left|{\mathrm{Value}}_{\mathrm{Real}}\right|\right|$$

(6) $$\mathrm{Mean}\;\mathrm{Error}\;=\;\frac{\sum_{i=1}^{\mathrm{Sample}\;\mathrm{Number}}{\left(\mathrm{Absolute}\;\mathrm{Error}\right)}_{i}}{\mathrm{Sample}\;\mathrm{Number}}$$

(7) Highest Error=3σ+Mean Error

The previous equations do not have any reference to describe the error and with this peculiarity, it cannot be clear how bad is that error. Thus, the full scale will be the reference with a value according to the peak to peak amplitude of the Redox current signal from the commercial potentiostat. The Mean Error Percent (MEP) describes how big the mean error is against the peak to peak amplitude as Equation (8) illustrates. The Highest Error Percent (HEP) describes how big this highest error is against the peak to peak amplitude as Equation (8) shows. Hence, these indicators will describe the error of the prototype measurements with a solid reference.

(8) $$\mathrm{Mean}\;\mathrm{Error}\;\mathrm{Percent}\;=\frac{\mathrm{Mean}\;\mathrm{Error}}{\mathrm{Peak}\;\mathrm{to}\;\mathrm{Peak}\;\mathrm{Amplitude}\;}\times 100$$

(9) $$\mathrm{Highest}\;\mathrm{Error}\;\mathrm{Percent}\;=\frac{\mathrm{Highest}\;\mathrm{Error}}{\mathrm{Peak}\;\mathrm{to}\;\mathrm{Peak}\;\mathrm{Amplitude}}\times 100$$

It is shown in Table 6 that the most relevant indicators for the error analysis. The MEP describes the error percent to expect in given measure. The HEP describes the maximum error percent to expect in an electrochemical trial. The CV errors are higher than those from the DSC and that can come from two factors: the full scale and the scan rate. However, it is difficult to know which of each has more weight because they are related. 

The experimental conditions 6 and 7 allow knowing the resolution of the equipment that can be related to the Lower Limit of Detection (LLD), thus in these trials the prototype measured the smallest signal value. From the conditions 6 and 7, it is possible to calculate the 5% of the mean error. Therefore, its values reflex the LLD to have an expected accuracy of 95% in the measurements compared with the commercial potentiostat. With that criterion, “*the prototype can handle currents above 86.44 nA and below of −86.44 nA,”* as Table 7 shows to have an accuracy above of the 95%.

**Figure 9:**
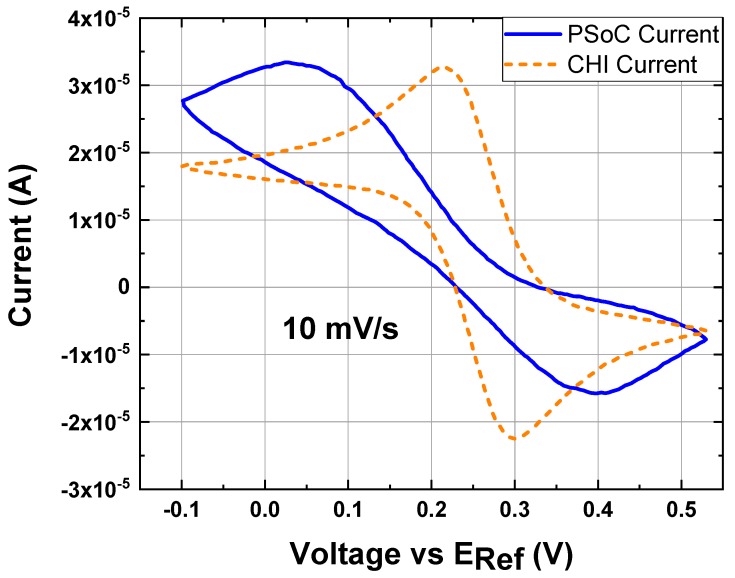
Cyclic voltammetry at 10 mV/s, 10 mM K_3_[Fe(CN)_6_] and 0.5 M KCl as the electrolyte support; the working electrode, the counter electrode and the reference electrode were a disk glassy carbon electrode (diameter ϕ = 3 mm), a platinum wire and Ag/AgCl electrode, respectively; experiment under the conditions 16: (Blue line) CV response by the EPS. (Orange line) CV response by the commercial potentiostat.

To obtain the Higher Limit of Detection (HLD) an additional experiment was carried out at the experimental conditions described in Table 8 (condition 16) and it is presented in Figure 9.

The HLD comes from the current values of the CV; specifically, condition 16 shows how the prototype cannot handle much current. The main difference between experimental conditions 1 and 16 is the concentration difference in the analyte (10 times in the order of magnitude). The Figure 9 describes how the EPS response is under a different phase than the commercial potentiostat. The condition one provides the HLD of ±3 µA according to the possible resolution of the system. However, trials with analyte concentration between (1 and 10) mM K_3_[Fe(CN)_6_] could prove a greater current range.

## 4. Conclusions

Table 9 shows the electrochemical conditions in which the response signal of the prototype is congruent with the commercial potentiostat. This table is a summary of the experiments to appreciate the capacity of the EPS. The concentration used provides information about the voltage and the current range of the electrochemical techniques studied. In addition, the study provides a guide to test the scan rate and the range of the sample per second on the EPS.

Out of the ranges of Table 9, the behavior of the EPS is erratic or unknown. The potentiostat loses the voltage control with analyte concentrations above 10 mM K_3_[Fe(CN)_6_]. As a result, the EPS capacity established in this study allows us to have several applications for the medical, biotechnology, environmental areas.

Table 10 describes the principal features of the EPS. The prototype has a capacity of ±2 V to control the voltage. The range of currents was established from the experiments discussed previously. The number of samples per seconds comes from the ADC selected in the PSoC. The scan rate range relies on the architecture of the PSoC and the algorithm that controls the waveform generator. The parameters described in Table 10 point out that the PSoC is a suitable device to work as a prototype of a portable EPS.

Table 11 and Table 12 give information about the power consumption and the values to compensate for having more accurate results. The offset voltages are summarized in Table 9 and the bias current at the inverting input establishes the minimum current to read in the device. The power consumption provides a clear idea of the battery requirements. 

### Potential Applications of the EPS Developed

According to Periasamy et al., it is possible to use their glucose biosensor in a linear concentration range of 6.3 to 20.09 mM [37] and the concentration can go from 2 to 22 mM in humans [38]. The biosensor has a sensitivity of 2.47 µA/(mM cm^2^). In addition, the output current range of the biosensor with a fixed area of 2 mm × 2 mm is of 0.62 to 1.98 µA. Hence, this prototype can handle that sensor because the EPS input current is wider than the biosensor output.

Apetrei et al. developed a sensor with a sensitivity of 37.1 nA/µM with an area of 0.867 cm^2^ in a linear range of 1–300 µM to detect melatonin [39]. With the EPS it is possible to detect melatonin in a concentration of 43 µM, since the detection range is around 2.5–80.0 µM. 

Other works have developed sensors with a sensitivity of 35 mA/(M cm^2^) for H_2_O_2_ [40,41,42]. In an amount of 10–200 µM, it can provoke a senescence-like state if a human cell gets in contact with it [42]. Thus, in a fixed area of 7.2 mm × 7.2 mm it is possible to detect it in a range of 5–165 µM with the EPS. 

Jaiswal et al. developed a biosensor for the determination of nitrite (NO_2_^−^) [43]. In that study, they found two linear ranges of 0.1 to 1 µM and 1 to 1000 µM having two different sensitivities of 1.25 µA/(µM cm^2^) and 0.005 µA/(µM cm^2^), respectively. As a result, the EPS can be useful in the detection of nitrite in a range of 0.1 to 833 µM with an electrode area of 0.72 cm^2^ and by considering the two slopes in this range.

Furthermore, the EPS can accomplish suitable features such as being a compact device, have a low power consumption, economically affordable, flexible for being programmed according to with the required necessity, suitable for being integrated over system-on-a-chip platforms, it provides accuracy in the range of measured currents. In addition, since there is a setup of slave-master on the EPS, then it becomes attractive to use this technology to install a network of different EPS to transmit via wireless communication the sensing data to the Potentiostat User Interface System (PUIS).

Finally, Table 13 presents a comparison of different compact potentiostats that have been studied to visualize their parameters in comparison to the parameters that can offer the EPS studied. 

Table 13 describes important features that should have the new generation in the electrochemical instrumentation, such as being small in size in order to be portable, economically affordable, precision in the measurements, low power consumption and wireless. The potentiostat designed, constructed and characterized in this work is demonstrated to be competitive with the previous work in potentiostats that has been shown recently (Table 13). Nonetheless, this work presented one of the first potentiostat constructed by using embedded electronics and it is the first of being a CYPRESS. For designing this potentiostat, the kits have a value of approximately cost (~100$ USD), therefore this approach to constructing a potentiostat can be very convenient versus other routes; also, the programmable circuit can vastly reduce the hardware complexity. Thus, it can lead the way to creating new applications for Point-of-Care with a reusable full electronics module. In addition, this work contributes to providing information about the architecture (digital peripherals and analog front end devices) required to construct a potentiostat since this information is scarce, due to the main providers being companies who protect their circuit design. Finally, an integral methodology that includes the characterization and calibration of the potentiostat has been presented, thus an analysis of errors on the measurements in this device were tested and three electrochemical techniques were performed. 

## Figures and Tables

**Table 1:** Sequences for the surface activation on the WE.

	Sequence 1	Sequence 2	Sequence 3	Sequence 4
Scan Rate	500 mV/s	250 mV/s	100 mV/s	50 mV/s
Cycles	50	25	10	5
Method	Cyclic Voltammetry			
Initial Voltage	0.25 V			
Maximum Voltage	0.65 V			
Minimum Voltage	−0.15 V			
Initial Scan Direction	anodic direction			

**Table 2:** Randles-Sevcik parameters and expected maximum current from CV and LSV experiments.

	Solution 1	Solution 2
K_3_[Fe(CN)_6_], analyte concentration	1 mM	10 mM
KCl, Electrolyte support concentration	0.5 M	
# of e^−^ per mole oxidized or reduced	1	
Analyte Diffusion Coefficient	7.23 µcm^2^/s [36]	
Scan Rate	10 mV/s	
Room Temperature	25 °C	
Surface Area of WE	0.071 cm^2^	
Randles-Sevcik Current Peak	5.11 µA	51.1 µA

**Table 3:** CV conditions for each experiment.

	Condition 1	Condition 2	Condition 3	Condition 4
Scan Rate	10 mV/s	100 mV/s	250 mV/s	500 mV/s
Initial Voltage	0.25 V			
Minimum Voltage	−0.15 V			
Maximum Voltage	0.65 V			
Recorded cycle for comparison	fifth			
Initial Scan Direction	anodic direction			
Analyte concentration	1 mM K_3_[Fe(CN)_6_]			
Electrolyte support concentration	0.5 M KCl			

**Table 4:** DSC conditions for each experiment.

	Cond. 5	Cond. 6	Cond. 7	Cond. 8	Cond. 9
First Step	0.325 V	0.315 V	0.305 V	0.295 V	0.195 V
Last Step	0.310 V				
Pulse Width	62 s				
Quite time	62 s				
Analyte	1 mM K_3_[Fe(CN)_6_]				
Electrolyte	0.5 M KCl				

**Table 5:** LSV conditions for the experiments.

	Cond. 10	Cond. 11	Cond. 12	Cond. 13	Cond. 14	Cond. 15
Initial Voltage	0.65 V	−0.15 V	0.65 V	−0.15 V	0.65 V	−0.15 V
Final Voltage	−0.15 V	0.65 V	−0.15 V	0.65 V	−0.15 V	0.65 V
Scan Rate	10 mV/s	10 mV/s	100 mV/s	100 mV/s	500 mV/s	500 mV/s
Analyte	1 mM K_3_[Fe(CN)_6_]					
Electrolyte	0.5 M KCl					

**Table 6:** Error analysis summary of the CV, DSC and LSV experiments in Figure 6, Figure 7 and Figure 8 and by using Equations (5–9). Experimental conditions are reported in Table 3, Table 4 and Table 5.

Experiment under the:	MEP, %	HEP, %	Mean Error, A	Highest Error, A	Full Scale, A	Method
Conditions 1	2.887	5.226	1.98 × 10 ^−7^	3.58 × 10 ^−7^	6.852 × 10 ^−6^	CV
Conditions 2	1.691	5.721	2.88 × 10 ^−7^	9.75 × 10 ^−7^	1.704 × 10 ^−5^	CV
Conditions 3	2.226	7.653	5.62 × 10 ^−7^	1.93 × 10 ^−6^	2.527 × 10 ^−5^	CV
Conditions 4	3.397	11.178	1.18 × 10 ^−6^	3.89 × 10 ^−6^	3.481 × 10 ^−5^	CV
Conditions 5	0.319	5.185	5.35 × 10 ^−9^	8.69 × 10 ^−8^	1.676 × 10 ^−6^	DSC
Conditions 6	0.587	5.386	3.6 × 10 ^−9^	3.30 × 10 ^−8^	6.134 × 10 ^−7^	DSC
Conditions 7	0.780	5.480	5.04 × 10 ^−9^	3.54 × 10 ^−8^	6.461 × 10 ^−7^	DSC
Conditions 8	0.562	6.169	1.03 × 10 ^−8^	1.13 × 10 ^−7^	1.827 × 10 ^−6^	DSC
Conditions 9	0.414	4.758	7.73 × 10 ^−8^	8.87 × 10 ^−7^	1.865 × 10 ^−5^	DSC
Conditions 10	2.019	5.994	9.55 × 10 ^−8^	2.83 × 10 ^−7^	4.728 × 10 ^−6^	LSV
Conditions 11	0.585	5.464	4.88 × 10 ^−8^	4.56 × 10 ^−7^	8.341 × 10 ^−6^	LSV
Conditions 12	2.837	8.429	3.12 × 10 ^−7^	9.26 × 10 ^−7^	1.098 × 10 ^−5^	LSV
Conditions 13	5.081	17.825	6.2 × 10 ^−7^	2.18 × 10 ^−6^	1.220 × 10 ^−5^	LSV
Conditions 14	4.243	14.483	9.94 × 10 ^−7^	3.39 × 10 ^−6^	2.342 × 10 ^−5^	LSV
Conditions 15	7.655	19.173	1.72 × 10 ^−6^	4.32 × 10 ^−6^	2.252 × 10 ^−5^	LSV

**Table 7:** Lower Limit of Detection analysis. *** Data did not evaluate in the study.

	Mean Error, A	LLD from 5% of the Mean Error, A
Conditions 6	3.6 × 10 ^−9^	***
Conditions 7	5.04 × 10^−9^	***
Conditions Average	4.32 × 10^−9^	86.44 × 10^−9^

**Table 8:** Condition to explore the highest limit of detection.

	Conditions 16
Scan Rate	10 mV/s
Initial Voltage	0.40 V
Minimum Voltage	−0.10 V
Maximum Voltage	0.53 V
Cycle	Fifth
Initial Scan Direction	Positive
Analyte	10 mM K_3_[Fe(CN)_6_]
Electrolyte Support	0.5 M KCl

**Table 9:** Electrochemical conditions evaluated. *** Data did not evaluate in the study.

	CV	LSV	DSC
Analyte Concentration	1 mM K_3_[Fe(CN)_6_]	1 mM K_3_[Fe(CN)_6_]	1 mM K_3_[Fe(CN)_6_]
Voltage Range	−0.15 to 0.65 V	−0.15 to 0.65 V	0.195 to 0.325 V
Peak Current Range	−3.0 to 4.0 µA	−2.5 to 3.5 µA	***
Maximum Step	***	***	0.310 to 0.195 V
Minimum Step	***	***	0.310 to 0.315 V
Maximum Current at 62 s	***	***	1.2 µA
Minimum Current at 62 s	***	***	43.22 nA
Scan Rate Range	10 to 500 mV/s	10 to 500 mV/s	***
SPS Range	50 to 2000 SPS	50 to 2000 SPS	50 to 2000 SPS

**Table 10:** Principal electric features of the EPS.

Voltage Range: ±2 V	Samples per Second: 50 to 2000
Current Range: ±3 µA	Scan Rate: 10 to 500 mV/s

**Table 11:** Additional features of the EPS with a source voltage of 5 volts.

ADC Offset Voltage: −61.056 µV	EPS consumption at Stand By: 137.5 mW
TIA Offset Voltage: −3.36034 mV	EPS consumption at 2000 SPS: 207.5 mW

**Table 12:** TIA resistor calculated and bias current at the inverting input.

TIA Resistors	Value Calculated	Bias Current at the Inverting Input
R1	18,971.46 Ω	3.40 × 10^−8^ A
R2	28,636.77 Ω	2.06 × 10^−8^ A
R3	38,272.14 Ω	1.67 × 10^−8^ A
R4	77,433.47 Ω	6.77 × 10^−9^ A
R5	116,828.09 Ω	5.35 × 10^−9^ A
R6	244,583.12 Ω	2.59 × 10^−9^ A
R7	490,370.41 Ω	1.27 × 10^−9^ A
R8	981,623.90 Ω	6.89 × 10^−10^ A

**Table 13:** Electrochemical Instruments Comparison. *** Data not available.

Electrochemical Instrument	Jafari et al. [44]	Dorta-Quinones et al. [45]	Bozorgzadeh et al. [46]	Dryden et al. [10]	EmStat 3+ Embedded/OEM [47]	WaveNow AFTP1 [48]	Sun et al. [11]	Giordano et al. [49]	Muñoz et al. (This Article)
Highest Current Detection Limit	350 nA	430 nA	950 nA	22 mA	100 mA	100 mA	200 µA	50 µA	3 µA
Lowest Current Detection Limit	8.6 pA	***	***	600 fA	1 pA	80 nA	1 nA	100 nA	86 nA
Electronic Chip Area	3 mm × 3 mm	1.5 mm × 1.0 mm	3.16 mm × 3.16 mm	***	***	***	***	***	***
PCB Area	***	4.7 cm × 1.9 cm	***	8 cm × 8 cm	5.5 cm × 4.1 cm	16.5 cm × 10 cm	3.9 cm × 1.62 cm	9.7 cm × 5.7 cm	~(8 cm × 6 cm)
Maximum Samples Per Second	2 ksps	10 ksps	10 ksps	30 ksps	Less than 1 Ksps	1 Ksps	200 ksps	***	2 Ksps
ADC Effective Number of Bits	9 bits	10.95 bits	***	21.3 bits at 1.45 ksps	***	***	***	***	18
Wireless Connectivity	Ultra-Wideband Transmitter	Ultra-Wideband Transmitter	FSK Transmission with Manchester Encoding	No	Bluetooth or Wifi	No	No	Bluetooth	Bluetootth
Number of Techniques Implemented	1	1	2	More than 3	9	33	3	4	At least 3
Channels	54	1	1	1	up to 16	1	2	1	1
SoC Present	Yes	Yes	Yes	No	***	***	No	No	Yes
Capabilities additional to a potentiostat	No	No	No	Yes	Yes	Yes	Yes	No	Yes, flexibility to reconfigure the analog front end
Maximum Power Consumption	1543.3 µW for each channel	30 µW	Approximately 0.4 mW	Less than 1 W	2.5 W	10 W	111 mW	***	207 mW at 5 V
